# *E2F1* germline copy number variations and melanoma susceptibility

**DOI:** 10.1186/s12967-019-1933-0

**Published:** 2019-05-29

**Authors:** Maria Santa Rocca, Clara Benna, Simone Mocellin, Carlo Riccardo Rossi, Aichi Msaki, Andrea Di Nisio, Giuseppe Opocher, Carlo Foresta

**Affiliations:** 1 Familial Cancer Clinic, Veneto Institute of Oncology (IOV-IRCCS), Padua, Italy; 2 Surgical Oncology Unit, Veneto Institute of Oncology (IOV-IRCCS), Padua, Italy; 3 Department of Medicine, Unit of Andrology and Reproductive Medicine, University of Padua, Via Giustiniani, 2, 35128 Padua, Italy; 4 Department of Surgery Oncology and Gastroenterology (DISCOG), University of Padua, Padua, Italy; 5 First Surgical Clinic, Azienda Ospedaliera di Padova, Padua, Italy

**Keywords:** *E2F1*, Copy number variations, Melanoma, Heat stress

## Abstract

**Background:**

Melanoma is an aggressive type of skin cancer whose aetiology remains elusive as both environmental and genetic factors can contribute to its development. Recent studies have demonstrated the existence of multiple copies of *E2F1* gene in melanoma specimens which could explain the deregulated E2F1 activity in this type of cancer. This finding suggests a key role for this transcription factor in the malignant transformation of melanocytes. Therefore, E2F1 has been considered as a potential therapeutic target for this form of skin cancer. Since germline copy number variations (CNVs) have been associated with increased susceptibility to different types of cancer, the aim of our study was to assess germline *E2F1* CNV in melanoma patients. However, CNVs not necessarily lead to gene dosage imbalance, hence, further factors, in association with CNVs, could contribute to clinical manifestations. Considering that heat stress has been hypothesised as a contributing factor to skin cancer, we also investigated the effect of heat stress on E2F1 expression.

**Methods:**

*E2F1* CNV was measured in genomic DNA isolated from blood of 552 patients diagnosed with melanoma and 520 healthy subjects using TaqMan Copy Number Assays. E2F1 mRNA expression was also evaluated by RT-qPCR in the melanoma cell line, SK MEL 267, before and after exposure to heat stress.

**Results:**

We found that patients diagnosed with melanoma (1.6%, 9/552) harboured frequently altered germline *E2F1* copies compared to healthy subjects (0%, 0/520). Moreover, the difference among the two groups was statistically significant (p = 0.004). Furthermore, we found that heat exposure alone can significantly induce E2F1 expression.

**Conclusions:**

This is the first study that shows a relation between germline *E2F1* CNV and melanoma, suggesting that altered copies of this gene might be a predisposing factor to skin cancer. Our results also suggest that environmental insults, such as heat stress, could contribute to an aberrant E2F1 activity by inducing E2F1 mRNA expression. Therefore, subjects with multiple constitutive copies of E2F1 are at greater risk of developing melanoma when exposed to heat. Altogether our results corroborate with the hypothesis that susceptibility to melanoma depends on both the environment and genetic factors.

## Background

Melanoma is the most deadly form of skin cancer that arises from uncontrolled proliferation of melanocytes. Since it grows and spread quickly, prompt diagnosis, surgery and treatment are necessary to prevent the development of metastases.

Despite remarkable progress in the last few years in the detection and treatment of melanoma, the underlying molecular mechanism that triggers the development of melanoma is still poorly understood [1].

Melanoma is a very complex and heterogeneous type of cancer whose main risk factors are: exposure to ultraviolet radiations (UVR), phenotypic traits and genetic alterations [2, 3].

Genetic anomalies account for approximately 5–10% of melanoma cases with an autosomal dominant inheritance pattern [4]. Acquired or inherited genetic mutations are not the only genetic factors to cause melanoma; also structural chromosomal abnormalities, gain or loss of specific genomic regions, can contribute to an aberrant gene expression in melanocytes [5, 6].

In melanoma specific set of duplicated portions of chromosome 20 have been observed [7–9] and, furthermore, most of the genes mapping on this chromosome were found to be upregulated in melanocytes in response to UV exposure [5]. The gene encoding for E2F1 transcription factor, which maps on chromosome 20, is found in multiple copies in both melanoma specimens and melanoma cell lines, resulting in the overexpression of the E2F1 protein [10]. E2F1 (UniprotKB: Q01094), belongs to the E2F family of transcription factor (TF), has a central role in regulating cell cycle progression and apoptosis [11, 12] and, therefore, E2F1 activity is tightly governed by multiple feedback mechanisms and by the tumour suppressor Rb. Given the pivotal role that E2F1 has in cell proliferation, its involvement in melanoma growth and progression is not surprising. In the last decade, germline CNVs in cancer-related genes, such as tumor suppressors or oncogenes, have been associated with cancer predisposition [13–15]. This strongly supports the amplification of *E2F1* gene in predisposing to cancer. Consistent with this, germline duplications of *E2F1* gene have been recently observed in testicular cancer patients, suggesting a potential role of *E2F1* copy number variations (CNVs) in the development of this type of cancer [16]. Whilst it has been established the somatic amplification of E2F1 in melanoma, germline *E2F1* copy number in melanoma patients has never been determined, in this study, we wanted to verify whether pre-existing CNVs of this gene might also predispose to melanoma.

Furthermore, although several genetic alterations have been proven to be reliable predictors of melanoma, to date UVR exposure is recognized as the major environmental risk factor for this cancer. However, the mechanism underlying melanocyte transformation induced by UV radiations is still not fully elucidated.

Calapre et al. [17] hypothesized that the upregulation of heat shock proteins (HSPs), provoked either by exposure to UVR or heat, or by the combination of both factors, may be responsible for skin cancer development by promoting cell survival and proliferation. Therefore, we also wanted to investigate whether heat stress alone may cause deregulation of key genes known to be involved in melanocyte malignant transformation.

## Methods

### Study design and patients enrolment

This study included a total of 552 Caucasian subjects (48.6% females and 51.4% males, median age at diagnosis of 53.8 ± 15.1 and 57.1 ± 14.9 years respectively) retrospectively selected among patients referred to the Veneto Institute of Oncology (IOV) for melanoma evaluation. Peripheral blood samples were stored in our institutional biobank (First Surgical Clinic—IOV) for genotyping purposes. As controls, 520 individuals (median age 40.2 ± 7.3) with no history of any malignancy were enrolled. All subjects provided written informed consent.

### Cell culture and reagents

The melanocyte cell line, SK-267-MEL, was grown in Dulbecco’s modified Eagle’s medium (Gibco; Gran Island, NY, USA) supplemented with 10% fetal calf serum, penicillin (100 U/ml) and streptomycin (100 U/ml). SK-267-MEL cultures were maintained in a humidified incubator at 37 °C with 10% CO_2_.

### Heat shock conditions

Heat shock was achieved by incubating cell cultures at 39 °C for 2 h in circulating water bath followed by 2-h recovery in an incubator at 37 °C. Control cells were cultured at 37 °C.

### DNA extraction

Genomic DNA was extracted from peripheral blood leucocytes of subjects and from melanoma cell line using QIAamp DNA Blood Mini Kit, according to the manufacturer’s protocol (Qiagen Inc., Hilden, Germany). The quality of the DNA was examined on a NanoDrop spectrophotometer (Thermo Fisher Scientific Inc, Waltham, MA, USA).

### Copy number variation analysis

Copy number variation was evaluated on 20 ng of genomic DNA. Quantitative real-time polymerase chain reaction (PCR) TaqMan Copy Number Assays were performed using three probes targeting different regions of the *E2F1* gene (Hs00576444_cn, Hs01758822_cn and Hs00919582_cn)(Applied Biosystems, Foster City, CA, USA). TaqMan CNV reactions were performed in triplicate using the FAM-dye-labeled assay for *E2F1* and VIC-dye labeled RNase P assay. Real-time data were collected by the StepOne Plus 2.1 software, and ABI CopyCaller 2.0 software (Thermo Fisher Scientific Inc, Waltham, MA, USA) was used for data analysis. Two independent assays were performed for each sample to confirm results.

### RNA Extraction, cDNA Synthesis and Real-Time PCR

Total RNA was extracted from SK-267-MEL using the RNeasy Mini Kit (Qiagen, Hilden, Germany). RNA was quantified by a NanoDrop spectrophotometer. cDNA was synthesized from 250 ng of total RNA retro-transcribed using SuperScript III (Invitrogen, Carlsbad, CA, USA) and random hexamers. Real Time PCR were performed in a 20 µl final volume containing 20 ng of cDNA, 1X Power SYBR Green PCR Master Mix (Applied Biosystem, Foster City, CA, USA), and a mix of forward and reverse primers (1 mmol/l each). The following primers were used: *E2F1*: forward 5′-CATCAGTACCTGGCCGAGAG-3′ and reverse 5′-CCCGGGGATTTCACACCTTT-3′; Heat Shock Protein 70 (*HSP70*): forward 5′-ATGAGTATAGCGACCGCTGC-3′ and reverse 5′-TCCTTGGACTGTGTTCTTTGC-3′. Human *GAPDH* was used as a housekeeping gene: forward 5′-TCGACAGTCAGCCGCATCTT-3′ and reverse 5′-AGGCGCCCAATACGACCAAA-3′. Real Time PCR was performed on thermocycler StepOne plus (Applied Biosystems, Foster City, CA, USA) using the following parameters: 95 °C for 10 min followed by 40 cycles of 95 °C for 15 s, 60 °C for 30 s, and 72 °C for 30 s. Relative quantification was performed using Delta Delta Ct (ΔΔCt) method (Livak KJ and Schmittgen, 2001). The qPCR products were verified melting curve and by agarose gel.

### Statistical analysis

Statistical analysis of the data was conducted with SPSS 21.0 for Windows (SPSS, Chicago, IL). Differences in the frequency of copy number variations between groups were compared using the Chi square test, or Fisher’s exact test when expected values were less than five.

Statistical power was calculated comparing two proportions: the frequency of *E2F1* altered copies in healthy controls and the frequency of *E2F1* altered copies in melanoma patients. The α level of significance was set at 0.05.

Results from qPCR were analysed using the two-tailed Student’s t test or the Mann–Whitney U test to determine statistical significance relative to exposed or non-exposed cells. A *p* value of ≤ 0.05 was considered statistically significant.

## Results

### E2F1 copy number variation in melanoma patients

Table 1 summarises the frequency of multiple copies of the *E2F1* gene in our cohort of 1072 subjects.

**Table 1:** Frequency of *E2F1* CNV in controls and melanoma patients

Subjects	CNV = 2	CNV > 2
Controls (N = 520)	520 (100%)	0 (0%)
Patients (N = 552)	543 (98.4%)	*9 (1.6%)*

CNV: copy number variation

Significance: values in italic refer to *p* = 0.004 vs Controls

All cancer free individuals had two copies of *E2F1* gene.

Although most of patients suffering from melanoma also harboured two copies of *E2F1*, a significant portion (1.6%, 9/552) had more than the two canonical copies of the gene. This was significantly higher than the frequency found within controls subjects (*p *= 0.004) (0%, 0/520) (Table 1). The statistical power of this study was 84%. Three independent TaqMan Copy Number Assays were performed to confirm this amplification of *E2F1* gene in the individuals with more than two copies of E2F1. Most of these subjects had an additional copy of the gene (Table 2). To understand whether the increased copy number of E2F1 gene impacted timing and site of melanoma manifestation, we collected the characteristics of the patients harbouring more than two copies of E2F1 gene. Table 2 lists the sex, number of *E2F1* copies detected, age at diagnosis and anatomical site of melanoma. The additional copy of *E2F1* would appear not to affect how soon and where melanoma develops in these patients, indeed, no correlation with gender, age and anatomical location of the primary malignant melanoma was detected.

**Table 2:** Characteristic of patients with *E2F1* CNV > 2

ID	Sex	Predicted CNV	Age (y) at time of 1st biopsy	Site of primary tumor
9025	F	4	64	Calf
16026	M	3	51	Underscapular back
16459	F	3	72	Arm
16461	F	3	60	Scapula
16464	F	3	66	Thigh
16541	M	3	47	Knee
16555	M	3	59	Back
16607	M	3	31	Scapula
19592	M	3	43	Arm

M: Male; F: female; CNV: copy number variation

### E2F1 expression analysis in a heat-stressed melanoma cell line

We used a melanoma cell line, SK-267-MEL, in order to determine whether environment insults such as heat stress may have an effect on the E2F1 expression. We performed also a TaqMan Copy Number Assay on SK-MEL-267 to investigate *E2F1* CNV and we found that this cell line carried two copies of the gene.

**Fig. 1:**
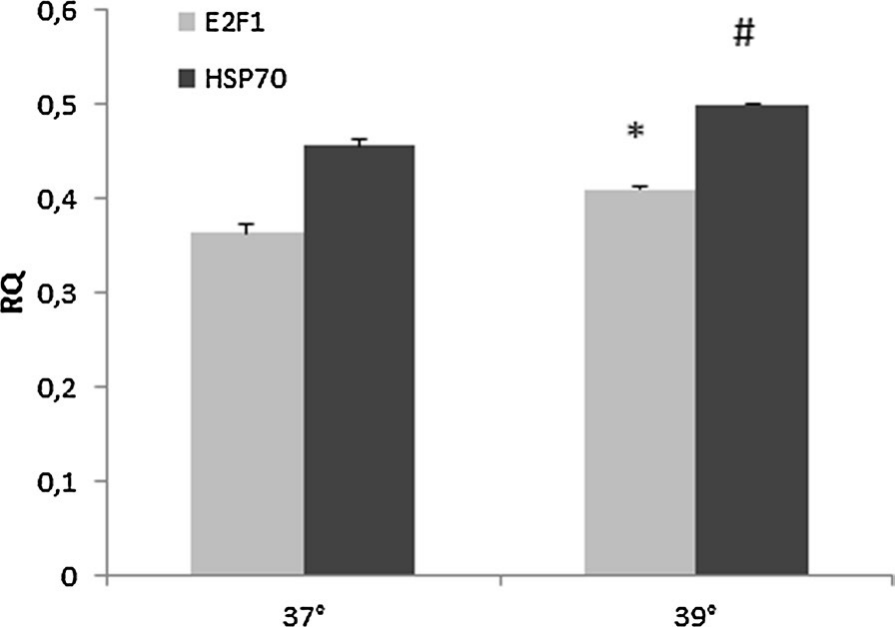
Quantitative real time of E2F1 and HSP70 mRNA in SK-267-MEL cells before and after heat shock. *p < 0.001 ^#^*p *< 0.0003

We exposed SK-267-MEL cells to heat shock and evaluated the mRNA expression of E2F1. In Fig. 1, the melanocyte cell line has an intact heat stress response as demonstrated by the upregulation of the heat shock protein HSP70 (p = 0.0003, Fig. 1). Critically heat also significantly induced the expression of E2F1 mRNA after only 2 h of exposure (*p *= 0.001, Fig. 1).

## Discussion

This is the first study showing an association between germline *E2F1* CNVs and melanoma development, suggesting a role for germline *E2F1* gains as a contributing risk factor for melanoma.

Moreover, this is the first study to demonstrate that genetic predisposition and environmental insults could act together to increase the risk of developing skin cancer. We demonstrate that heat stress could directly induce the mRNA expression of E2F1 in melanoma.

The most recognized environmental risk factor for melanoma are UVR as they cause formation of reactive oxidative species which severely damage cells and cause gene mutations. Mutations of *CDKN2A* gene account for roughly 20% of melanoma cases [18]. Furthermore, the deregulation of pRb pathway due to *CDKN2A* or *CDK4* gene mutations is particularly frequent in melanomas arising from skin chronically exposed to sun [8]. The control of cell cycle entry is one of the most tightly controlled event that protects cells from uncontrolled proliferation and genomic instability. E2F1 regulates the G1/S entry by transcribing several genes necessary for DNA replication and cell cycle progression. In quiescent cells, E2F1 is inhibited by the Rb protein, which upon mitogenic stimulation is phosphorylated and releases E2F1. Several oncogenes inactivate Rb and loss of the E2F1 negative feedback system allows the uncontrolled proliferation of cancer cells. The deregulated activity of E2F1 protein could also be the result of chromosomal rearrangements involving *E2F1* gene, such as CNV encompassing *E2F1* gene, as demonstrated by this and our previous studies (Table 1 and ref. [16]). We previously reported germline gains of *E2F1* in men with testicular cancer (13). We found that individuals harbouring constitutive duplications of this gene are more likely to develop this form of cancer and to be infertile [16]. Alarmingly, overexpression of E2F1 protein was found in the testicular tissue of a testicular cancer patient who had multiple germline copies of *E2F1* (13). Even though it remains to be further elucidated which molecular pathways are involved in the upregulation of E2F1 protein, this finding strongly suggests a contribution of germline *E2F1* copy numbers in rendering this individual particularly susceptible to cancer of the testis. In addition to an increased risk for testicular cancer, subjects with altered germline *E2F1* copies are potentially at risk of developing also melanoma, since *E2F1* was also found in multiple copies in many melanoma cases [10]. Testicular cancer has been, therefore, associated with an increased risk for melanoma [19], suggesting that these two cancers share common environmental and genetic factors.

In this study we report that germline gains of *E2F1* also occur in a relevant number of melanoma patients (Table 1). These gains were absent in individuals not affected by melanoma, whereas all subjects with multiple copies of *E2F1* had melanoma. These results imply that the acquisition of additional germline copies of *E2F1* could predispose to melanoma later in life.

Although it is undeniable that E2F1 acts as oncogene in several cancers, including in melanoma, to the extent that it has been proposed as a new therapeutic target [20], it is not clear which molecular mechanism triggers the transcription of *E2F1* and consequently lead to the neoplastic transformation of melanocytes. Our recent discovery of E2F1 expression induced by heat [21], suggests that this condition, might be the one context in which this genetic alteration could cause the overexpression of E2F1 protein and its activity leading to the uncontrolled proliferation of melanocytes.

Heat stress has been, furthermore, recently indicated as potential risk factor for skin cancer, since it could trigger the transcription of HSPs in melanocytes, promoting cell proliferation and survival [17]. The detrimental effect of temperature has been already reported also in other form of cancers, such as oesophageal cancer, where chronic heat exposure leads to tumorigenesis [22]. Interestingly, in this study we demonstrate that heat stress stimulate the expression of E2F1 mRNA in melanoma cell line which further supports its role as potential risk factor for melanoma.

However, CNV amplification does not necessarily lead to overexpression of the gene encoded within the CNV. We hypothesize that stress conditions, such as heat, could trigger the transcription and consequently the translation of E2F1 protein. Indeed, considering that one genetic alteration is generally insufficient to induce melanoma, it is likely that the combination of environmental and genetic factors are necessary for the onset of melanoma.

## Conclusion

This study suggests a potential role of CNV in tumorigenesis as we found an association between germline *E2F1* CNV and melanoma, however, further studies are needed in order to determine whether the additional *E2F1* copy was acquired de novo or inherited. Furthermore, information on the lifetime exposure to high temperature of the subjects would also be necessary to understand the impact that it might have had on the timing and site of melanoma manifestation.

The identification of downstream targets of heat inducing E2F1 activity in melanoma could provide further insight on the underlying molecular pathways that lead to the development of this cancer.

## Abbreviations

UVR: ultraviolet radiation
CNV: copy number variation
TF: transcriptional factor
HSP: heat shock protein

## Availability of data and materials

All data generated or analysed during this study are included in this published article.
